# High-grade glioma in very young children: a rare and particular patient population

**DOI:** 10.18632/oncotarget.18478

**Published:** 2017-06-14

**Authors:** Moatasem El-Ayadi, Marc Ansari, Dominik Sturm, Gerrit H. Gielen, Monika Warmuth-Metz, Christof M. Kramm, Andre O. von Bueren

**Affiliations:** ^1^ Department of Pediatrics and Adolescent Medicine, Division of Pediatric Hematology and Oncology, University Hospital of Geneva, Geneva, Switzerland; ^2^ Department of Pediatrics, CANSEARCH Research Laboratory, Faculty of Medicine, University of Geneva, Geneva, Switzerland; ^3^ Division of Pediatric Neurooncology, German Consortium for Translational Cancer Research (DKTK), German Cancer Research Center (DKFZ), Heidelberg, Germany; ^4^ Department of Pediatric Oncology, Hematology, and Immunology, Heidelberg University Hospital, Heidelberg, Germany; ^5^ Institute of Neuropathology, University of Bonn Medical Center, Bonn, Germany; ^6^ Reference Center for Neuroradiology, University Hospital of Wuerzburg, Wuerzburg, Germany; ^7^ Division of Pediatric Hematology and Oncology, University Medical Center Goettingen, Goettingen, Germany; ^8^ Department of Pediatric Oncology, National Cancer Institute, Cairo University, Cairo, Egypt

**Keywords:** infants, brain tumors, high-grade glioma, chemotherapy, radiotherapy

## Abstract

In the past years, pediatric high-grade gliomas (HGG) have been the focus of several research articles and reviews, given the recent discoveries on the genetic and molecular levels pointing out a clinico-biological uniqueness of the pediatric population compared to their adult counterparts with HGG. On the other hand, there are only scarce data about HGG in very young children (below 3 years of age at diagnosis) due to their relatively low incidence. However, the few available data suggest further distinction of this very rare subgroup from older children and adults at several levels including their molecular and biological characteristics, their treatment management, as well as their outcome. This review summarizes and discusses the current available knowledge on the epidemiological, neuropathological, genetic and molecular data of this subpopulation. We discuss these findings and differences compared to older patients suffering from the same histologic disease. In addition, we highlight the particular clinical and neuro-radiological findings in this specific subgroup of patients as well as their current management approaches and treatment outcomes.

## INTRODUCTION

Central Nervous System (CNS) tumors are the second most common cancers affecting children and adolescents after leukemia. Approximately half of these CNS tumors are childhood gliomas. Unlike in adult patients, the majority of childhood gliomas are low-grade gliomas (LGG), whereas high-grade gliomas (HGG) account for approximately 8-12% of all primary CNS tumors in children [1], with the most frequent types being anaplastic astrocytoma (WHO grade III) and glioblastoma (WHO grade IV) [2].

Treatment strategies have been similar for children and adolescents with HGG compared to adults. However, outcomes remain dismal with long-term survival rates around 10% [2, 3]. Recent knowledge in the biology, molecular and genetic characteristics of these tumors implies that pediatric HGG comprise one or most probably several distinct entities that might be treated differently based on their genetic and epigenetic features [4].

There are only scarce data available about HGG in children younger than three years of age due to their very low incidence in this age group. Reported data suggest that very young children with HGG might have a better prognosis compared to older children or adults [3, 5–7]. This might be – at least partly - explained by differences in molecular and biologic characteristics [8].

Here, we aim to review the current knowledge of very young children with HGG (here –in agreement with most pediatric neuro-oncology working groups- defined as patients younger than three years) regarding their epidemiology, genetic and epigenetic characteristics as well as their clinical management and treatment outcomes.

## EPIDEMIOLOGY

Very young children differ from older children and adolescents regarding incidence and location of different histological entities of CNS tumors [1, 9]. Around 10% of primary CNS tumors occur during the first year of life with almost half of them during the first six months. About 18% of these tumors appear before the age of two years [10].

In a recent publication from the Central Brain Tumor Registry of the United States (CBTRUS), the highest overall incidence of childhood CNS tumors was in infants (below one year of age; 6.22 per 100,000 children) followed by ages 1 – 4 years (5.53 per 100,000 children) [1]. Earlier data from Surveillance, Epidemiology, and End Results (SEER) data-base for the period between 1973 and 2006 report on a much lower overall incidence of CNS tumors in infants (3.1 per 100,000) [11].

In a meta-analysis of data from 16 studies, the most frequent histological entity in pediatric CNS tumors was astrocytoma (37.6%) [12]. According to data from 60 countries published by the International Agency for Research on Cancer (IARC), annual incidence rate for patients with astrocytoma ranged between 0.7 – 2.2 per 100,000 children [13].

Regarding infant CNS tumors, Bishop et al. examined the SEER registry and found that gliomas had the highest incidence among infants with a rate of 1.38 per 100,000 [11]. Similarly, CBTRUS reports gliomas as the most common histological entity in infants (37.2% of tumors) and in children aged 1 – 4 years (58.1% of tumors). The majority of these gliomas were LGG (62.5% and 61.2%, respectively) and the highest incidence for HGG (26%) was observed in children aged 5 – 9 years [1]. In contrast, a German case series from 1984 to 2000 reported HGG as the most frequently occurring in very young children (17.7%) and in adolescents aged 15 – 17 years (21.7%) [12].

The annual incidence rates for CNS tumors in children as well as the frequency of different histologic diagnoses are largely variable among individual studies [14, 15]. In the pediatric oncology group (POG) experience, HGG were the 4th most common malignant brain tumor in very young children following medulloblastoma, ependymoma and primitive neuro-ectodermal tumors [16]. Interestingly, two thirds of these HGG occurred in infants younger than 6 months at diagnosis [16]. Similarly, the children's cancer group (CCG) experience of HGG shows same tendency for clustering early in life with 21 out of 32 patients aged less than one year at diagnosis [6]. On the other hand, the French BBSFOP and United Kingdom UKCCSG 9204 studies didn't show similar trend, where patients younger than 1 year comprised only 7 out of 21 patients and 6 out of 19 patients, respectively [5, 17]. In addition to true ethnic, geographical and socio-economic differences, this variability between reports is likely due to differences in patients’ numbers, variable age cut-offs, as well as the pathological classification system followed in each study. Table 1 summarizes the incidence of HGG in childhood in different age groups as reported by several studies and registries. However, the incidence of individual diagnoses might change in the future, as the pathological classification is continuously edited and modified to be complemented by novel molecular tests and characteristics.

**Table 1:**
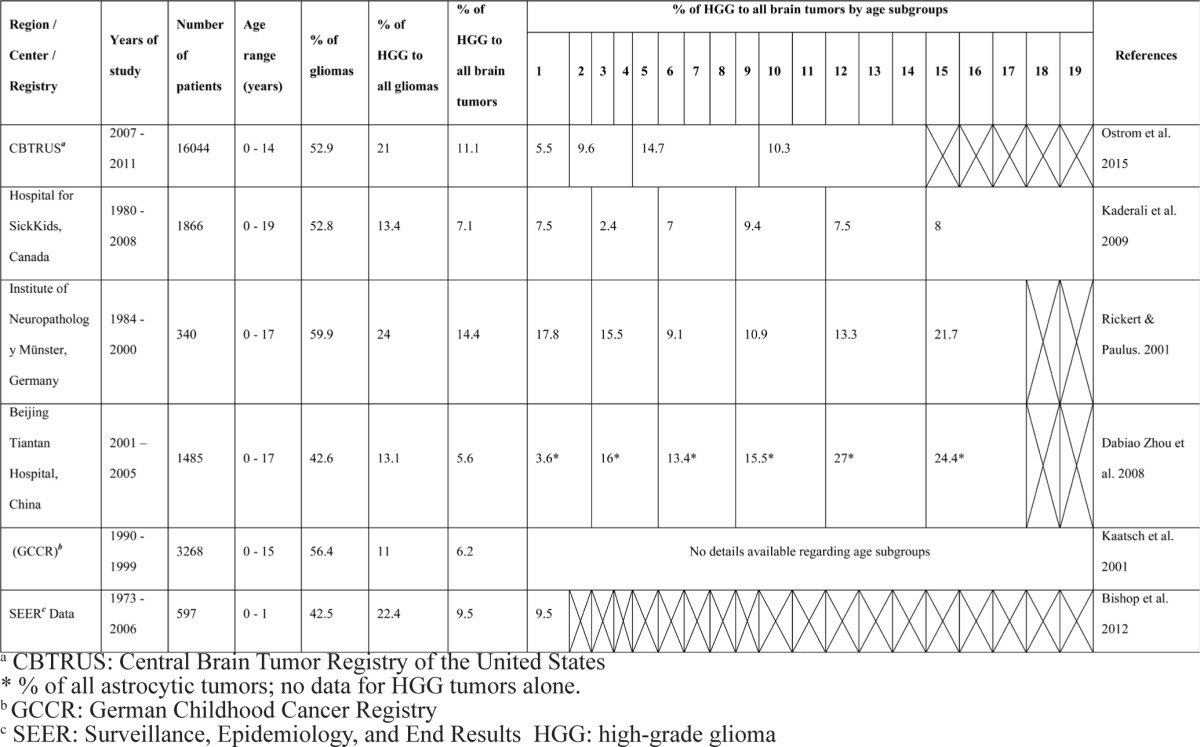
Incidence of childhood HGG in different age groups as reported by selected studies

HGG in very young children typically occur in hemispheric locations and less frequently in posterior fossa or other midline locations [18, 19]. This is evident in the POG, CCG and UKCCSG 9204 studies where 83%, > 50% and 90% of HGG were located in cerebral hemispheres, respectively [6, 16, 17].

## PATHOLOGICAL DIAGNOSTICS

**Figure 1:**
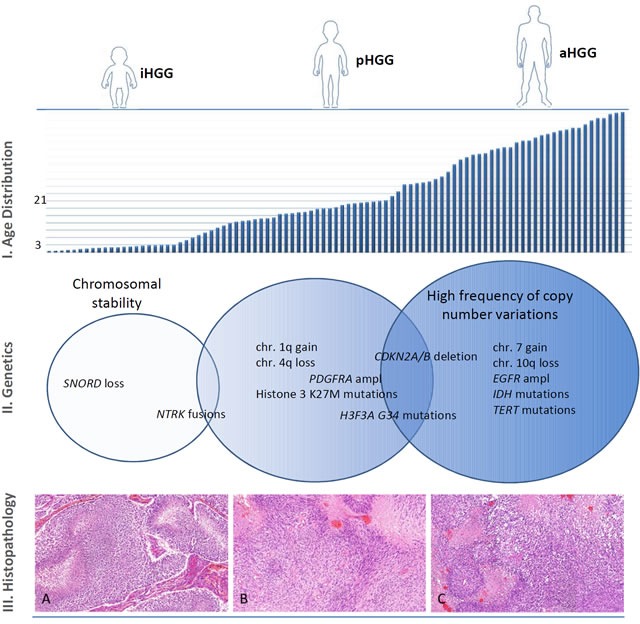
Age distribution, genetic abnormalities and histopathological findings of HGG Diagrammatic illustration demonstrating the age distribution, genetic abnormalities and histopathologic findings of HGG in very young children, older children and adults. I. Age Distribution: Bar graph showing increasing incidence of high-grade glioma depending on age at diagnosis. Of notice, the age cutoff definition for HGG in very young children varies between different groups (from 2 - 5 years), but most pediatric neuro-oncology working groups define it at three years of age. Similarly, definition of age cutoff for pediatric HGG patients varies between different groups and ranges from 16 to 21 years. II. Genetics: HGG in very young children tend to display more stable genome and few identifiable mutations, with SNORD loss and NTRK fusion genes being the most common molecular abnormalities. HGG in older children are characterized by increasing frequency of copy number aberrations, like PDGFRA amplification and CDKN2A/CDKN2B deletions and/or histone 3 (K27M or G34R/V) mutations. Adult HGG frequently display copy number variations like gain of chromosome 7 and loss of chromosome 10q as well as EGFR amplification, IDH and TERT mutations. III. Histopathology: H&E stained tumor specimens of glioblastoma in **a.** a very young child, **b.** an older child and **c.** an adult patient. All three examples share histological high-grade features with cell-rich tumor areas, microvascular proliferation and pseudo-palisading necrosis as well as a high mitotic activity. A histology-based discrimination of these tumor samples allowing conclusion regarding the patient's age is impossible.

Histology-based diagnosis is still the most reliable, fastest and cost-effective diagnostic approach in brain tumor classification, even though the most recent 2016 WHO classification of Tumors of the Central Nervous Systems[20] introduces multi-layered, i.e. histological and molecularly based definitions of brain tumors entities. In conventional histological and immunohistochemical staining, HGG in very young children do not show differences compared to pediatric or adult HGG (Figure 1). Routine diagnostic tools of formalin-fixed and paraffin-embedded tumor samples of diffusely infiltrating HGG should at least include a hematoxylin and eosin (H&E) staining and a reticulin silver staining as well as immunohistochemical examination with antibodies against glial fibrillary acidic protein (GFAP), p53 and Ki67 (Mib-1). Map2, Olig-2 and ATRX can be of use, while IDH-1 (R132H) immunohistochemistry does not play a role in diagnosis of HGG in very young children. Diffuse midline gliomas harboring histone H3 K27M mutations correspond to WHO grade IV, even though typical high-grade features (e.g. microvascular proliferation, necrosis, increased proliferative and mitotic activity) may be absent. Glial tumors arising in midline CNS structures should therefore always be tested for histone H3 mutations, possible also by a mutation-specific H3 (K27M) antibody. For HGG entities of much lower incidences (pediatric type anaplastic oligodendroglioma (WHO grade III), anaplastic (pilocytic) astrocytoma (analogue WHO grade III), pleomorphic xanthoastrocytoma with anaplastic features (WHO grade III) and anaplastic ganglioglioma (WHO grade III), additional immunohistochemical and molecular examination is often necessary. As a putative diagnostic pitfall especially in infant cases, the presence of desmoplastic infantile astrocytoma and ganglioglioma (DIA/DIG), corresponding to WHO grade I, should be taken into consideration, since these entities can present as large tumor masses with sometimes a poorly differentiated tumor cell component, possibly mimicking high-grade features.

## GENETIC AND EPIGENETIC FEATURES

Recent studies on the molecular features of pediatric HGG highlighting different genetic and epigenetic alterations strongly suggest a clear distinction from their adult counterparts [21–23] and they defined different subgroups in terms of cell of origin, clinical characteristics and prognosis [4]. Due to their rarity, HGG in very young children are still understudied and molecular insights are limited.

In this section we discuss the few available data on molecular characterization of HGG in very young children within the context of main molecular findings in pediatric HGG (Figure 1).

### Structural abnormalities: Copy-number alterations and focal aberrations

Pediatric HGG exhibit broad chromosomal abnormalities at a much lower frequency compared to adult HGG, with chromosome 1q gains being the most common aberration while 7q gain and 10q loss are less commonly encountered [24,25].

Paugh et al. observed recurrent 1q gains in 29% of cases and focal *platelet-derived growth factor receptor α (PDGFRA)* amplifications in 12% of tumors in their report on 78 pediatric HGGs [25]. Other studies reported higher frequency of *PDGFRA* amplifications up to 39% of pediatric HGG [26, 27]. Unlike adult HGG, *PDGFRA* amplifications in pediatric HGG had no prognostic significance [27]. However, a recent study by Koschmann et al. reported worse outcome in correlation with *PDGFRA* mutations and not amplifications [28]. Other amplifications such as *epidermal growth factor receptor (EGFR)*, *MYC*, *MYCN* and *MDM4* are encountered in 1–4 % of tumors. The most frequent copy-number losses are 10q (38%), 13q (34%) and 14q (29%) [25].

In a recent study investigating the molecular features of 35 HGG from very young children, cytogenetic alterations were in general less frequent compared with HGG from older pediatric and adult patients but included some alterations which are commonly seen in HGG (gains of 1q and 7q or losses of 10q, 13q and 14q) [8]. Gain of 1q was seen in 22.7%, loss of 6q in 18.2%, and 10q loss in 9.1% of cases. Losses of 13q and 14q as well as focal amplifications of *PDGFRA* or *EGFR* were absent, while *cyclin dependent kinase inhibitor -2A (CDKN2A)* deletions were seen in two cases. Genomic alterations were more frequently seen in children older than nine months, which might imply an association between older age at diagnosis and accumulation of genetic alterations [8]. These findings are supported by results of Paugh et al. who included 11 very young children in their study cohort of pediatric HGG. In those very young children, 1q gain was not detected and 10q loss was observed in one case [25].

Loss of *SNORD* genes coding for small nucleolar RNAs (snoRNAs) has been previously reported in several types of cancer [29–32]. Recently, it was detected in 27.3% of HGG in very young children [8]. This finding raises some questions regarding the role of snoRNAs in gliomagenesis in HGG in young children and their potential uses as diagnostic markers or as novel therapeutic targets.

One of the common findings in pediatric HGG is the expression of fusion genes, yet with most of them being non-recurrent fusions. A study by Wu et al. identified recurrent fusion genes involving the kinase domain of the *neurotrophin tyrosine receptor kinase* gene (*NTRK*) in 4% of brainstem and 10% of non-brainstem HGG, being observed in 40% (4/10) of HGG in very young children [26]. Recently, recurrent *NTRK* fusion genes were reported in adult HGG as well as pediatric LGG [33, 34], and later shown to induce HGG in mice by injection of astrocytes transduced with retroviral vectors carrying *NTRK* fusion genes [26]. The authors suggested a significant oncogenic effect of *NTRK* fusion genes in very young children based on their high frequency in that age group, lack of other significant mutations, as well as rapid tumor induction in their animal model [26].

### Somatic histone mutations

Detection of recurrent somatic histone H3 mutations is one of the major discoveries emphasizing the unique nature of pediatric HGG [35, 36]. Specific mutations in *H3F3A* coding for H3.3 and *HIST1H3B/C* coding for H3.1 were recently recognized in pediatric HGG as the first reported histone mutations associated with human malignancies [35, 36].

Initial reports identified two distinct single-nucleotide variant mutations, methionine replacing lysine 27 (K27M) frequently involving *H3F3A* and to a lesser extent *HIST1H3B,* and arginine (or less frequently valine) replacing glycine 34 (G34R/V) in *H3F3A* [35, 36]. These mutations were found in more than one third of cases of pediatric HGG in a mutually exclusive pattern. When other gliomas of different grades and across all ages were examined for these mutations, they were identified exclusively in HGG with the vast majority of them occurring in children and adolescents [23, 35, 36]. The anatomical tumor locations and age distribution of patients for each group of histone mutations differed largely. All G34R/V mutated tumors were found in non-midline cortical locations (hemispheric) and mainly affect young adults and adolescents. K27-mutated cases were found in midline tumors (thalamus, cerebellar vermis, brainstem, and spine) and affect younger children (∼ 10 years of age) [35–37].

In contrast, Gielen et al. found *H3F3A* K27M mutations only in two cases of their cohort of very young children with HGG (6%). The authors suggested that the difference in frequency might be attributed to the anatomical distribution of tumors in their cohort. Only five cases – including those two with *H3F3A* K27M mutations – were midline tumors, while the majority of patients (30/35, 86%) had supratentorial hemispheric tumors [8]. However, the authors did not report *H3F3A* G34R/V mutations in any of the thirty hemispheric tumors. Thus, anatomical distribution alone might not adequately explain the different frequency of these mutations among very young children with HGG.

Schwartzentruber et al. found marked overlap of *TP53* mutation (more than half of cases) with other mutations in *H3F3A*, the *alpha thalassemia/mental retardation syndrome X-Linked* (*ATRX*) gene, and the *death-domain-associated protein* (*DAXX*) gene [35]. Loss of ATRX protein expression in their samples was strongly associated with alternative lengthening of telomere (ALT), particularly in cases with simultaneous *ATRX*, *H3F3A* and *TP53* mutations. This finding is in agreement with an earlier study of Heaphy et al. who reported a strong association of ATRX protein loss and ALT [38]. Conversely, Liu et al. failed to prove such an association in both adult and pediatric HGG [39]. As for HGG in very young children, Gielen et al. found loss of ATRX protein expression in only six cases (17%) and a similar frequency of ALT but with only minimal overlap, suggesting that other mechanisms for telomere lengthening maybe present in HGG of very young children [8]. However, 40% of their cases had either loss of ATRX protein expression or *H3F3A* mutation or p53 accumulation, pointing to the importance of the *H3F3A/ATRX/DAXX* pathway in a subgroup of HGG in very young children [8].

### DNA methylation profiling

Genome-wide studies investigating global DNA methylation in HGG identified different subgroups in adult and pediatric HGG [23], of which three subgroups included most pediatric patients. One subgroup, receptor tyrosine kinase 1 (RTK-I), was characterized by predominant *PDGFRA* amplification and included patients from a wide age range. The other two subgroups correspond to the two *H3F3A* mutations (K27M and G34V/R). Among adolescents and young adult HGG, one of the identified subgroups was strictly related to *IDH1* mutations [23].

Korshunov et al. investigated the DNA methylation profiles of more than 200 pediatric HGG. They found almost 20% of histologically confirmed HGG showing methylation patterns similar to LGG (LGG-like) and pleomorphic xanthoastrocytoma (PXA-like). The majority of those cases were of cortical location (around 75%), had better overall outcome (5-year OS > 90% for LGG-like and ∼ 60% for PXA-like cases) and frequently harbored *BRAF-V600E* mutations (48% in PXA-like and 31% in LGG-like). Of interest, very young children with HGG comprised 54% of LGG-like cases and 11% of PXA-like cases in their retrospective study [40].

In summary, HGG in very young children tend to display a more stable genome with less frequent CNAs and fewer identifiable mutations.

## CLINICAL PRESENTATION AND DIAGNOSIS

The clinical presentation of a newly diagnosed child with a brain tumor depends on tumor location, and on the age rather than the underlying histology [41]. Different brain tumors share common initial symptoms of increased intracranial tension plus or minus focal deficits and other localizing symptoms according to their anatomical location [42]. Notably, anatomical distribution of brain tumors varies between very young children and their older counterparts with prevalent supratentorial locations in very young children [43, 44].

The onset of symptoms in very young children with a brain tumor is often delayed due to the elasticity and expandability of infants’ skulls that allow tumors to grow significantly before manifesting with increased intracranial pressure [45, 46]. The symptoms they develop in such young age are often non-specific and are usually attributed to other etiologies leading to further delay in diagnosis. These symptoms include macrocephaly, nausea and vomiting, irritability, lethargy and failure to thrive [41, 47–49].

For any child with suspected intracranial tumor, a magnetic resonance imaging (MRI) is the diagnostic standard. It helps identifying tumor location, defining its relation to surrounding structures and more importantly guiding further interventions like surgical resection and radiation field planning [50]. Non-contrast CT scan is particularly useful in emergency situations when acute hydrocephalus or CNS vascular accidents are suspected [50].

**Figure 2:**
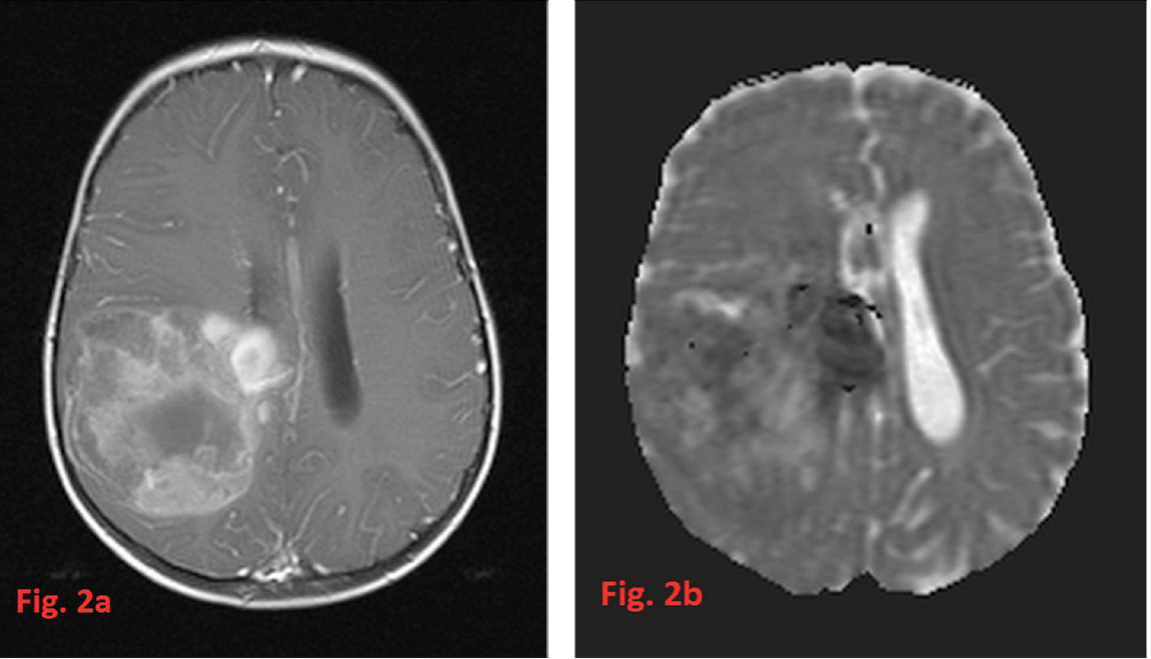
In a right frontoparietal large glioblastoma; enhancement on a T1-weighted MRI after contrast (**a**) is very inhomogeneous. The corresponding ADC-map (**b**) also shows a variability of restricted diffusion with the most restricted areas above the roof of the right lateral ventricle. On T1-weighted images before contrast (not shown) also met-hemoglobin as residue of subacute bleeding is present.

**Figure 3:**
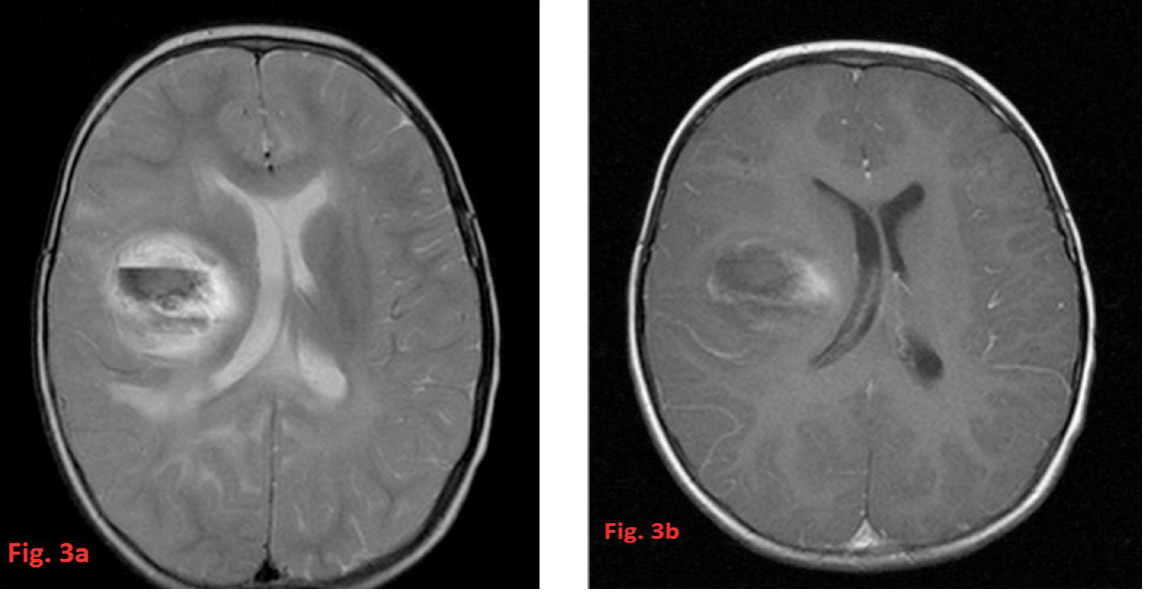
T2-weighted axial image (**a**) showing a central hemorrhagic part (with a fluid level) of a thalamic glioblastoma together with an increased T2-signal in the posterior corpus callosum, the right frontal region and the left frontobasal parts (not shown) of the brain as a result of multifocal growth. The T1-weighted image at the corresponding level after contrast (**b**) shows only slight enhancement surrounding the central deoxyhemoglobin core of this tumor part and very ill defined borders.

HGG tend to appear as ill-defined, irregularly shaped tumors with heterogeneous textures and nodular enhancement. This presentation often prevents a measurability of tumor size especially after surgery when postoperative changes blur the tumor margins additionally. They are usually accompanied by significant brain edema best evidenced on Fluid attenuation inversion recovery (FLAIR) and T2 sequences. They frequently display hypo-intense signals on T1 images and hyper-intense signals on T2 images [51]. Only rarely the T2-signal of the tumor is heavily decreased due to a very high cellularity comparable to embryonal tumors. In this case the imaging differential diagnosis to e.g. ependymomas may be difficult. Most HGG show intense contrast enhancement, however, the degree of contrast enhancement is not always consistent with histopathologic grading [51]. Figures 2 and 3 show some radiological findings of HGG in very young children.

New imaging techniques have been lately developed for better prediction of the underlying tumor grade, including magnetic resonance spectroscopy (MRS) that works through analyzing the metabolic profile of tumor which reflects its grade [51–53]. Similarly, perfusion-weighted MRI can detect increased tumor blood flow caused by angiogenesis in high grade tumors [51, 54]. Diffusion-tensor MRI calculates an apparent diffusion coefficient (ADC) reflecting tumor cellularity and hence tumor grade [55]. All these modalities still need further validation of their predictability and accuracy. None of them can replace histopathologic examination of tumor tissue in order to reach a definite final diagnosis [50].

Around 3% of pediatric HGG are associated with dissemination into cerebrospinal fluid (CSF) at presentation [56]. Spread usually occurs with tumors related to ventricles or meninges where they can seed directly into the subarachnoid space [57]. The incidence of dissemination in very young children with HGG is quite variable among different studies; while the POG experience shows a much higher incidence with 4 out of 18 children having leptomeningeal dissemination at presentation, both the French BBSFOP and United Kingdom UKCCSG 9204 studies had no cases with CSF-dissemination. However, the very small numbers of patients in each of these studies preclude an accurate estimation of the real incidence of the disease dissemination in this very young patient subpopulation. Although leptomeningeal dissemination and distant spread are relatively uncommon in HGG patients [2, 42], it is usually recommended to perform a spinal MRI and CSF examination at time of diagnosis and whenever there is a suspicion for a relapse and/or dissemination [42, 56, 58]. Of interest, no survival advantage has been reported for non-disseminated gliomas compared to those with CSF dissemination [16, 57].

## TREATMENT OF HIGH-GRADE GLIOMAS IN PEDIATRIC POPULATION

Over the past decades, several clinical trials have been conducted on pediatric HGG patients aiming for improving treatment outcome of this devastating disease, but unfortunately, little progress has been achieved [2, 3, 59–64]. Fewer trials have been conducted on very young children with HGG [5, 6, 16, 17], and despite their relatively better outcome compared to older children, no much progress has been achieved over the past decade since the last published trials [5, 17].

### Surgery

As a general rule, whenever safe and feasible, gross total resection (GTR) of the tumor should be attempted in every child with HGG. However, in many occasions a safe complete tumor resection cannot be achieved [50]. This is particularly true for midline and infratentorial tumors that comprise –unfortunately - a major proportion of pediatric HGG cases [1]. The majority of HGG in very young children are supratentorial hemispheric tumors, and hence expected to be more amenable to GTR, yet this is not achievable in most patients. Despite marked decline in surgical mortality rates for very young children with malignant brain tumors, surgical morbidity is still a major issue [65]. Most cases present with enormous tumor sizes (tumor brains)[66] and are frequently associated with massive hydrocephalus, both of which cause marked distortion of brain vasculature, thus increasing the risk of intra and post-operative hemorrhage [65, 67]. In addition, given their relatively small blood volume, very young children are more vulnerable to hypovolemic shock and cardiac arrest due to blood loss during surgery [65, 67]. In most series, GTR was achievable in approximately one third of cases, and was reported in 6 out of 17 evaluable patients in the POG study [16], 7 out of 21 patients in the French series [5], 14 out of 32 patients in the CCG trial [6], and 3 out of 19 patients in the UKCCSG 9204 study [17].

In a recently published, relatively small single-center experience from Japan, neoadjuvant multi-agent chemotherapy was utilized for infants and very young children with brain tumors to facilitate tumor resection by reducing the vascularity of the tumor [68]. Eight out of 9 patients had GTR following neoadjuvant chemotherapy and only 3 of them required blood transfusion with no surgical mortality reported [68].

Generally, extent of resection is considered as a powerful prognostic factor that impacts survival outcome in HGG patients. Adults with completely resected HGG tumors appear to have a survival advantage [69]. Although patient numbers may be smaller, pediatric HGG patients appear also to benefit from GTR of the tumor with an improvement of survival [70–73]. In a report from the study-945 by the Children's Cancer Group (CCG), pediatric HGG children with GTR (≥ 90%) had better 5-year progression-free survival (PFS) compared to those with lesser extent of resection (35±7% and 17±4%, respectively) [74].

However, this finding cannot be extrapolated to the very small population of very young children with HGG. Whereas some analyses showed evidence for a better survival after GTR of the tumor [16, 75], other studies could not confirm this survival advantage [5, 6]. These inconsistent findings might be -at least partly- explained by small patient numbers in each of these individual studies. Table 2 illustrates different studies and clinical trials for very young children with HGG. As residual tumor/disease appears to be associated with less favorable prognosis, a second surgery might be considered during the course of the treatment. In addition, Jeibmann et al. reported on a case of infant HGG that showed a differentiation towards low grade histology upon chemotherapy [76]. Thus, residual tumor after induction chemotherapy might be subjected to a second look surgery - even if only a partial resection or a biopsy can be performed - with the aims to increase the probability of survival and/or to avoid any second line treatment, e.g. radiotherapy, since this residual may no longer represent a HGG-like histology.

**Table 2:** Summary of the different studies in infants and very young children with primary high-grade gliomas

Reference	No. / age of pts	Treatment regimen	Extent of resection	Radiotherapy	Treatment results	Neuropsychologic sequelae
Duffner et al. 1993, USA Multicenter trial (POG)	< 3 years of age n=18	2 cycles Cyc/VCR followed by 1 cycle Cis/Eto, to be repeated for 1 or two years until radiotherapy can be performed at age > 3 years	Better prognosis after gross total tumor resection (6/18)	14/18 received radiotherapy according to the protocol	1 y PFS 54% ; OS 83% 2 y PFS 54% ; OS 65% 3 y PFS 43% ; OS 50%	No difference in cognitive functions evaluation between base-line and after 1 year of chemotherapy
Geyer et al. 1995, USA Multicenter trial (CCG)	< 2 years of age n=32 (20 AA, 3 AOA, 8 HGG, 1 GS)	“Eight drugs in 1 day”: VCR, BCNU, Procarbazine, Hydroxyurea, Cis, ARA-C, DTIC, Pred. Potential RT after 10 cycles and/or PD	No better prognosis after gross total (14/32) and subtotal (9/32) tumor resection	4/32 in total with 2 at relapse	3 y PFS : Total 31%, AA 44%, HGG 0%	Not evaluated
Dufour et al. 2006, France Multicenter trial (BBSFOP)	<5 years of age n=21 (4 AO, 5 AOA, 7 AA, 5 HGG)	Alternating cycles with Carbo/Procarbazine, Eto/Cis, VCR/Cyc	No better survival after gross total tumor resection (7/21)	6/21 at relapse/ progression	5 y PFS 35.3%, 5 y OS 58.8% No significant survival differences between grade III and IV tumors	For all evaluable survivors (n=7); the mean full scale intellectual quotient was 81.6 (range 55–104).
Sanders et al. 2007, USA Single Center Study	<3 years of age n=15 (9 AA, 5 HGG, 2 malignant glioma)	Different chemotherapy protocolsa with 6 patients receiving scheduled radiotherapy during treatment	Better OS, but no better EFS after gross total and near total tumor resection (11/16).	12/15 in total with 6 at relapse/ progression	5 y EFS / OS Total 28.6%, 66.3%%, AA 33.3%, 77.8%, HGG 40% 80%	Evaluable survivors (n=9) with some neurocognitive impairment, overall cognitive ability ranging from average to significantly delayed
Thorarinsdottir et al. 2007, USA Single Center Study	<4 years of age n=5 (1 AO, 1 AGG, 2 AA). Brainstem glioma, n=1 (AA)	3 cycles of induction Cis/Cyc/VCR/Eto followed by 3 cycles HDC with Carbo / Thiotepa and ASCR	2/5 with 1 gross total and 1 near total tumor resection	3/5 after high dose chemo-therapy	1 dead; PFS & OS 17 & 22 months. 1 alive with progressive disease; PFS & OS: 3 & 10 months. 3 alive without progression; PFS/OS 8, 33 & 59 months.	All evaluable survivors (n=4) with a normal cognitive development status
Grundy et al. 2010, UK Multicenter trial UKCCSG CNS9204,	<3 years of age n=18 (7 AA, 2 AO, 1 AAB, 5HGG, 3 unknown / unclassified)	Alternating courses of: Course 1: VCR / Carbo Course 2: VCR / MTX Course 3: VCR / Cyc Course 4: Cisplatin	3/18 total resection with only 1 long-term survivor, 11/18 partial tumor resection	5/18 at relapse/ progression	1-Y EFS 52.6%, OS 57.9% 3-Y EFS 24.1%, OS 40.5% 5-Y EFS 18.1%, OS 34.7%	Not mentioned
Mason et al. 1998, USA multicenter Study (Head Start 1)	<6 years of age n=9 4 / 9 were < 3 years of age	5 cycles of induction VCR / VP16 / Cis / Cyc followed by consolidation Carbo / thiotepa / VP16 with aBMT in 2 patients only	2 GTR, 5 STR. For all diagnoses, GTR had better prognosis in survival time from diagnosis but not OS after aBMT	5 / 9 due to progression after induction chemotherapy	1-Y EFS 11%, OS 56% 2-Y EFS 11%, OS 22%	Mean scores for un-irradiated children were within average range for academic achievement; verbal learning; visual memory, social-emotional, and behavioral functioning.
Razzouk et al. 1995, USA multicenter Phase II Study	< 4 years of age n=4	2 induction cycles of Thiotepa (TT) followed by alternative cycles of (Cyc/VCR), (Cispat/VP16) and (TT)	Not detailed	2 / 4 due to progression after induction chemotherapy	b None had an objective response to induction with TT; 2 PD, and 2 SD	Not mentioned

Abbreviations: AA = anaplastic astrocytoma WHO III; AGG = anaplastic ganglioglioma WHO III; AO = anaplastic oligodendroglioma WHO III; AOA = anaplastic oligoastrocytoma WHO III; AAB = anaplastic astroblastoma WHO III; ARA-C = cytosine arabinoside; carbo = Carboplatin; CCG = Children's Cancer Group; Cis = cisplatinum; Cyc = cyclophosphamide; DTIC = dacarbazine; EFS = event-free survival; Eto = etoposide; HGG = glioblastoma multiforme WHO IV; GS = gliosarcoma WHO IV; HGG = high-grade glioma; OS = overall survival; PFS = progression-free survival; POG = Pediatric Oncology Group; Pred= prednisone; RT = radiotherapy; VCR = vincristine

a Different chemotherapy regimens reported by Sanders et al. (2007): MOPP (n=1; Nitrogen Mustard/VCR/Procarbacine/Pred); CNS2 (n=1; Cis/Eto); Baby POG (n=4; VCR/Cyc alternating with Cis/Eto); CNS6 (n=1, upfront window with 2 cycles Thiotepa followed by Cis/Eto alternating with VCR/Cyc); CNS11 (n=2; VCR/Cyc alternating with Cis/Eto); CNS14 (n=5, Cyc/Carbo/Eto); BB98 (n=2; 2 cycles VCR/Cyc/intrathecal Mafosfamide alternating with oral Eto)

b Unacceptable high rate of Progressive Disease ♢early termination of trial

### Radiotherapy

In 1979, Walker et al. showed that post-surgical radiotherapy of glioblastoma significantly improved median survival time from 4-5 to 9-12 months [77]. However, vulnerability of the brain to cancer therapies – particularly radiation therapy - poses a challenge when therapeutic options are being considered. Children aged < 3 years are more vulnerable to the radiotherapy-induced sequelae when compared to older children [16, 78, 79]. These sequelae include neuropsychological and cognitive problems, endocrinopathy, vasculopathy with stroke, and secondary malignancies [78–82].

In order to minimize these damaging sequelae, chemotherapy-based therapies were utilized to defer or delay radiotherapy until the age of 3 years [5, 6, 16, 17]. Pediatric oncology group (POG) and CCG studies were designed to offer radiation therapy –at a reduced dose - for all patients at the end of chemotherapy, however, many patients in their cohorts did not receive any radiation therapy and were treated by chemotherapy alone [6, 16]. In four out of 18 infants with HGG treated in the Baby POG trial, radiation therapy was denied by their families and – interestingly - they were all alive at the end of the study and none of them experienced any recurrence for several years later [10]. In the CCG study, only four out of 32 patients received radiotherapy with two of them getting irradiation following disease relapse. Out of the four irradiated patients, only one – none relapsing – patient remained alive by the end of study [6].

Both the French BBSFOP and United Kingdom UKCCSG 9204 studies were restricting irradiation to the time of relapse or tumor progression only. This restriction did not seem to compromise the overall survival of studied cohorts [5, 17]. In the French cohort, out of 21 HGG patients below 5 years of age, 12 patients were still alive at the end of study, of whom 10 patients received no radiotherapy [5]. The UKCCSG 9204 study reported a cumulative radiotherapy rate of 29% at 5 years for HGG patients within their cohort, supporting the assumption that chemotherapy alone appears to be an adequate first-line treatment for very young children with HGG [17]. On the other hand, the experience from the St. Jude Children's Research Hospital for 16 very young children with HGG suggested that radiotherapy was essential with 12/16 patients receiving irradiation whether as a part of their initial treatment or at time of disease progression/relapse [75]. However, it should be noted that this experience was based on a single center retrospective study and not a prospectively controlled trial as in other studies (Table 2).

### Chemotherapy

Chemotherapy was established as an effective and essential element for the treatment of pediatric HGG when the CCG phase III clinical trial (CCG-943) showed for the first time that adjuvant radio-therapy plus chemotherapy (prednisone, chloroethyl-cyclohexyl nitrosourea [CCNU], and vincristine) significantly improved event-free survival (EFS) in children and adolescents with HGG compared to children and adolescents treated by radio-therapy alone (5-year EFS of 46% and 18%, respectively) [60]. Since then, multimodal treatment regimens for pediatric HGG patients with adjuvant chemotherapy was generally accepted. Design and treatment results of large trials for the treatment of children and adolescents with HGG are listed in Table 3.

**Table 3:** Design and results of large international multicenter trials for the treatment of children and adolescents with high-grade gliomas

Clinical Trial	Reference	Chemotherapy and/or targeted therapy	Radio-therapy	Key results	Special Remarks
CCG-943	Sposto et al. 1989	VCR weekly concomitant to RT, thereafter 8 cycles in 6-week intervals with Pred, CCNU, VCR (pCV) vs. RT alone	54 Gy	46% 5y EFS (chemo+radio) vs. 18% (RT alone); significant only for HGG patients not for AA.	Retrospective neuropathological review showing many LGG
CCG-945	Finlay et al. 1995; Fouladi et al. 2003	pCV chemotherapy plus radiotherapy (standard treatment) vs. 2 cycles experimental treatment with “8-in-1” chemotherapy (VCR, CCNU, procarbazine, hydroxyurea, cisplatinum, cytarabine, dacarbazine, methylprednisolone) upfront RT, no concomitant chemotherapy to RT, 8 additional courses “8-in-1” after RT	54 Gy	33% 5y PFS, 36% 5y OS. No difference in survival between standard and experimental treatment. Corrected for centrally confirmed HGG: 5y PFS 19%, OS 22%	29% of patients with LGG after consensus neuropathology review
CCG-9933	MacDonald et al. 2005	Pre-irradiation chemotherapy with either 4 courses of Cyc/Eto or of carbo/Eto or of Ifos/Eto. All these treatment regimens were followed by RT with concomitant VCR and maintenance treatment with VCR/CCNU	54 Gy	8% 5y-EFS, 24% 5y-OS. No significance in survival between the different upfront chemotherapy groups.	Randomized phase II pre-irradiation window study for non-completely resected HGG
POG-9135	Finlay and Zacharoulis 2005	Pre-irradiation Cis/BCNU vs. pre-irradiation VCR/CyC	54 Gy	20% 5y-PFS for Cis/BCNU, 5% PFS for VCR/Cyc	Randomized phase III pre-irradiation window study
POG-9431	Chintagum-pala et al. 2006	Pre-irradiation chemotherapy with either 2 courses of procarbazine or of topotecan. This treatment was followed by RT with concomitant VCR as well as maintenance treatment with VCR / CCNU	54 Gy	10% 3y-EFS, 15% 3y-OS. No significant differences in survival between the different upfront chemotherapy groups.	Randomized phase II pre-irradiation window study for non-controlled trial with historical control
HIT-HGG A	Wolff et al. 2000, 2002	21 day courses of oral Eto and trofosfamide with a subsequent rest for one week in parallel to RT and after RT for up to one year	54-59 Gy	4.3% 4y-EFS, 21.7% 4y-OS for non-brainstem HGG. 0.05% 4y-OS for DIPG	Non-controlled trial with historical control
HIT-HGG B	Wolff et al. 2006	One cycle of Cis, and Eto at the beginning and one of Cis, Eto, and Ifos at the end of RT followed by maintenance treatment with daily interferon-γ and 3 weekly Cyc	54-59 Gy	18,3% 2y-OS for non-brainstem HGG 0% 2 y OS for DIPG	Non-controlled trial with historical control
	Bronischer et al. 2009	Escalating daily doses of erlotinib in parallel and after RT for a planned maximum of 3 years.	54-59 Gy	2y-OS 485, 2y-PFS 35%; HGG: 1y-OS 67%, 1y-PFS 33%; AA: 1y-OS 86%, 1y-PFS 75%.	Clinical phase I trial
	Geyer et al. 2010	Escalating daily doses of gefitinib in parallel and after RT for up to one year.	55.8 Gy	For non-brainstem HGG: 1y-OS 28.8%, 1y-PFS 15.4%. For BSG: 1y-OS 48%, 1y-PFS 16.1%.	Clinical phase I trial
HIT-HGG C	Wolff et al. 2008, 2010	One cycle of Cis, Eto, and VCR at the beginning of RT followed by weekly VCR and then one cycle of Cis, Eto, and Ifos at the end of RT, thereafter repeated every 4 weeks up to week 29 as first maintenance. After week 29 valproic acid as second maintenance until progression.	54-59 Gy	For DIPG and non-brainstem HGG: 56% 1y-OS / 30% 2y-OS / 19% 5y-OS; 27% 1y-EFS / 16% 2y-EFS / 13% 5y-EfS. BSG: Median OS 1.13 y, median EFS 0.40y.	Non-controlled trial with historical control
HIT-HGG D pilot	Wolff et al. 2011	Upfront 2 courses of HD MTX, then RT with 1 cycle of Cis, Eto, VCR at the beginning, weekly VCR during RT and 1 cycle of Cis, Eto, Ifos. at the end, Maintenance with VCR / CCNU / pred q 6 weeks for up to 8 cycles.	54-59 Gy	For DIPG and non-brainstem HGG: 77% 1y-OS / 40% 2y-OS / 13% 5y-OS; 43% 1y-EFS / 20% 2y-EFS / 13% 5y-EfS.	Non controlled pilot trial with historical control
ACNS 0126	Cohen et al. 2011a, b	Radio-chemotherapy with daily TMZ followed by TMZ maintenance for 5 days every 28 d for 10 cycles	54-59 Gy	Non-brainstem HGG: 3y-OS 22%, 3y-EFS 11%. HGG: 3y-EFS 7%; AAIII: 3y-EFS 13%.	Non-controlled trial with historical controls

Abbreviations: AA = anaplastic astrocytoma WHO III; BSG = brainstem glioma; Carbo = carboplatin; Cis = cisplatinum; Cyc = cyclophosphamide; EFS = event-free survival; Eto = etoposide; HGG = glioblastoma multiforme WHO IV; HGG = high-grade glioma; OS = overall survival; PFS = progression-free survival; Pred= prednisone; RT = radiotherapy; VCR = vincristin

Starting in the 1980s, different chemotherapy regimens were examined in the early “baby brain studies” for all types of brain tumors in very young children. Several chemotherapeutic agents were tried in different combinations that could be given for long periods to replace or postpone radiotherapy [5, 16, 83, 84]. Many of these studies suggested the effectiveness of chemotherapy alone to sustain long-term survival in very young children with HGG [5, 6, 16, 17]; for details see Table 2.

Response of post-operative measurable disease to initial chemotherapy varied between different studies. In the Baby POG study, 10/18 very young children with HGG had less than GTR and six of these 10 patients showed partial response after two cycles of chemotherapy [16]. The CCG trial of the 8-in-1 chemotherapy that included 32 infants with HGG did not show similar response rate. Only five out of 21 patients (24%) with measurable post-operative residual tumor showed partial response to 2 cycles of chemotherapy [6]. The BBSFOP study included 13 out of 21 patients with post-operative residual tumor [5]. Only 2 out of 13 patients showed partial responses to chemotherapy. In their analysis, age seemed to have a prognostic impact – though statistically non-significant – with 6 out of 11 children less than 2 years were long-term survivors compared with 2 out of the 10 patients older than 2 years [5].

Similar to studies of older children with HGG, variability in histopathologic classification of tumors was also evident among many studies of HGG in very young children. Six of 26 patients with malignant astrocytoma in the UKCCSG/SIOP CNS-9204 trial were found to have a different diagnosis following central pathological review [17] while 18% of infant HGG patients in the CCG-945 trial were reclassified after central review [6,10]. Similarly, 4 out of 16 patients (25%) in the St. Jude experience had revised diagnoses after pathologic review [75]. In contrast, 20 /21 patients in the BBSFOP study had same initial diagnosis after central review by a panel of four neuropathologists. Of notice, they reported one third of their cases as having oligodendrogliomas, a much higher percentage than those reported by other trials [5].

### High-dose chemotherapy and autologous stem cell rescue

The potential role of high-dose chemotherapy (HDCT) followed by autologous stem cell rescue (ASCR) was previously investigated for very young children with CNS tumors including HGG [85–87]. Initially, it was mainly utilized for recurrent and progressive brain tumors [88, 89]. The Head Start 1 protocol investigated the efficacy of HDCT followed by ASCR for very young children with newly diagnosed brain tumors [90]. Sixty-two patients below 6 years of age – with 37 patients of them below 3 years of age - were enrolled including 9 patients with non-brainstem HGG. Only 37 patients were non-progressing after induction chemotherapy and hence received myeloablative chemotherapy followed by ASCR. Of those 37 patients, only 15 patients were long-term survivors without evidence of progression. Only 2 patients with HGG received HDC and ASCR and they were both alive and disease-free at end of study. The 3-year EFS and OS for all patients were 25% and 40%, respectively with no difference in outcome between the group of patients < 3 years of age and those > 3 years of age at diagnosis [90].

Tandem HDCT/ASCR treatment was investigated in children younger than 4 years old with newly diagnosed brain tumors [91]. Fifteen patients – including 5 patients with HGG – received induction chemotherapy followed by 3 cycles of HDCT (thiotepa / carboplatin) with ASCR. Their results were relatively better with 2-year PFS and OS of 52.2% and 72.1%, respectively. Of notice, 7 out of the 15 patients received selective radiotherapy after ASCR, however, the PFS of those who received irradiation did not differ significantly from those who did not. Ten out of the 15 patients –including 3 patients with HGG – were still alive and free of disease at end of study [91].

## CONCLUSION

HGG in very young children are relatively rare tumors that are challenging to treat, yet they are characterized by a better prognosis compared to HGG in older children. Whether this better outcome when compared to older patients might be due to difference in biology of the disease, needs further investigations. The relation between age at diagnosis and accumulation of genetic alterations needs further exploration to help determine the best age cut-off that distinguish this particular group of tumors from their counterparts in older children. These tumors in very young children appear to be sensitive to chemotherapy and sometimes might be cured without irradiation. However, no advances of treatment strategies have been achieved in the past few years. More collaborative efforts are needed to decipher the molecular and genetic landscape of these rare tumors, to identify new potential therapeutic targets, and hence to move forward in the management of this disease in these very young patients.
